# *Lactobacillus*‐Derived Biosurfactants: Emulsification and Antioxidant Properties in Oil Emulsions

**DOI:** 10.1002/fsn3.70334

**Published:** 2025-06-05

**Authors:** Roghayeh Rezaeimalidareh, Mohammad Ahmadi, Seyed‐Ahmad Shahidi

**Affiliations:** ^1^ Department of Food Science and Technology Am.C., Islamic Azad University Amol Iran; ^2^ Department of Food Hygiene Am.C., Islamic Azad University Amol Iran

**Keywords:** oxidation, PCR identification, surface activity, traditional yogurt

## Abstract

This study aimed to extract biosurfactants from *lactobacilli* present in traditional yogurt and evaluate their emulsifying and antioxidant performance in emulsions formed with various oils. First, yogurt samples were cultured in MRS medium, resulting in the isolation of 19 isolates (N1–N19). Morphological analyses and chemical tests on the colonies showed that seven isolates belonged to the *Lactobacillus* genus. Next, hemolysis, oil spread, drop collapse, and emulsification index tests were conducted. The results indicated that none of the isolates exhibited hemolytic activity; however, isolates N4 and N18 demonstrated good performance in oil spread (8.1 ± 0.16 cm and 7.4 ± 0.04 cm, respectively) and droplet collapse (droplets with diameters larger than 1 cm) tests, also showing high emulsification index (42.49 ± 0.81 and 39.05 ± 0.32, respectively) at this stage. The two isolates, N4 and N18, were identified using PCR techniques and were named *Lactobacillus plantarum* LBp_WAM and *Lactobacillus rhamnosus* DSA LR1, respectively. Biosurfactant extraction and purification were then performed on these strains, and emulsification index, emulsion stability, and creaming index tests of the biosurfactants were conducted on emulsions formed with olive, canola, sunflower, and rice bran oils. The results showed that the biosurfactants had high emulsification index, although their effect was less than that of Tween 80. Finally, the assessment of oxidation levels indicated that the emulsions prepared with biosurfactants were significantly less oxidized compared to the control sample. The findings of this research introduce the biosurfactants derived from *L. plantarum* and *L. rhamnosus* as natural biosurfactants for emulsion preparation, particularly with olive and canola oils.

## Graphical abstract

Extracted biosurfactants from *lactobacilli* present in traditional yogurt had emulsifying performance in emulsions. Oxidation levels indicated that the emulsions prepared with biosurfactants were significantly less oxidized compared to the control sample. This research introduces the biosurfactants derived from *L. plantarum* and *L. rhamnosus* as natural biosurfactants for emulsion preparation, particularly with olive and canola oils.

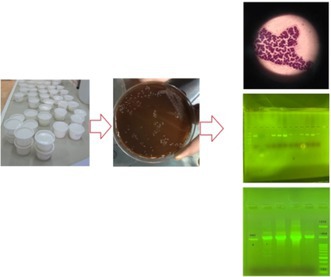

## 1 Introduction

Surfactants are amphiphilic compounds that contain both hydrophobic and hydrophilic segments. These substances have been employed in the food industry for centuries to reduce surface tension between two immiscible phases and to facilitate the formation of emulsions (Kralova and Sjöblom 2009; Sakr et al. 2021). Based on their origin, surfactants can be classified into synthetic surfactants and biosurfactants (Abdel‐Rahem 2024; Moldes et al. 2021). Synthetic surfactants are synthesized through organic chemical reactions, whereas biosurfactants are produced by microorganisms (De et al. 2015; Hajfarajollah et al. 2018). Biosurfactants have several distinct advantages over synthetic surfactants, including higher biodegradability, lower toxicity, and higher activity under extreme temperatures, pH, and salinity (Miao et al. 2024). These characteristics have garnered significant attention from the scientific community as they may serve as promising candidates to replace several synthetic surfactants. Biosurfactants can be used in several different industrial sectors such as food, cosmetics, petroleum, etc. In the food industry, biosurfactants can be used as formulation ingredients due to their ability to reduce surface tension and stabilize emulsions. In other industries, biosurfactants can be used to increase the apparent solubility of chemical contaminants, prepare laundry detergents, extract crude oil, increase the transport of crude oil through pipelines, clean oil storage tanks, treat oil waste, and prepare sustainable formulations of cosmetics and skin and hair care products (Drakontis and Amin 2020).

The primary source of biosurfactants is bacteria, and in this context, lactic acid bacteria (LAB) are recognized as prominent biosurfactant producers among probiotic bacteria (Echresh et al. 2024; Hajfarajollah et al. 2018). Lactic acid bacteria are typically described as a group of gram‐positive, non‐spore‐forming, catalase‐negative, fully fermentative, and acid‐tolerant bacteria that produce lactic acid as the main product of their fermentative metabolism. Lactic acid bacteria play crucial roles in the fermentation of dairy and meat products and significantly influences the quality and preservation of final products (Coelho et al. 2022). Among food‐related LAB, the genus *Lactobacillus* possesses numerous beneficial characteristics that make it suitable for industrial applications (Behbahani and Noshad 2024; Goyal et al. 2012; Rahmati‐Joneidabad et al. 2024).

*Lactobacilli* exist in various forms, ranging from long and narrow bacilli to short coccobacilli (Behbahani et al. 2024; Seddik et al. 2017). These gram‐positive, facultatively anaerobic, non‐motile, non–spore‐forming bacteria ferment glucose into lactic acid and provide a protective effect in preserving fermented foods due to their production of organic acids and the lowering of pH, resulting in extended shelf life and improved quality of fermented food products (Actor 2012). They can withstand weak acids with a pH of 3.5–4.5, leading to the production of 90% lactic acid (Goyal et al. 2012). This bacterium was first isolated from milk, and today its presence in various fermented foods, meats, vegetables, beverages, and confections has been established (Gharehyakheh et al. 2019; Zhang et al. 2023).

Yogurt is a popular fermented dairy product produced by LAB such as *Streptococcus thermophilus* and *Lactobacillus delbrueckii bulgaricus* . During the production of yogurt, these bacteria produce lactic acid, which causes the coagulation of milk proteins (Nagaoka 2019). Various studies have been conducted regarding the isolation of *Lactobacillus* species from yogurt, indicating that different species of *Lactobacillus* may be present in yogurt. Roushan Zadeh et al. (2014) isolated *L. acidophilus* , *L. fermentum* , *L. plantarum* , *L. helveticus* , and *L. delbrueckii* from various yogurt samples (Roushan Zadeh et al. 2014). Recently, Hajimohammadi Farimani et al. (2016) isolated and identified *L. plantarum* from yogurt and milk samples from different regions of Khorasan Razavi (Hajimohammadi Farimani et al. 2016). According to the conducted research, some of these species are capable of producing biosurfactants and can be utilized in the food industry as biosurfactants. Recently, multi‐functional emulsifiers have garnered considerable attention. For instance, these emulsifiers exhibit antioxidant and antimicrobial activities (Behbahani et al. 2024; Ghelichi et al. 2023).

The emulsification properties and the impact of microbial biosurfactants on reducing the oxidation of perishable products encourage researchers to explore the characteristics of novel and stable biosurfactants with high potential for emulsion stabilization and protection of essential fatty acids in food emulsions from oxidation (Hosseinian‐Roudsari et al. 2022; Keivanfar et al. 2023; Sheybani et al. 2023; Zibaei‐Rad et al. 2024). Given the importance of producing biosurfactants from biological sources, there has been no research conducted on the production of biosurfactants by *Lactobacillus* species from dairy sources in Mazandaran Province, Iran, despite the studies carried out regarding the isolation of biosurfactants from dairy sources. Therefore, this study aimed to evaluate the performance of biosurfactants produced by *Lactobacillus* isolated from traditional yogurt in various oils.

## 2 Materials and Methods

### 2.1 Materials

Traditional yogurt was purchased from savadkuh in Mazandaran Province, Iran. The refined olive, canola, sunflower, and rice bran oils, without any antioxidants, were purchased from Ghoncheh Oil Refinery factory (North Cultivation and Industry Co., Iran). All chemicals, culture media, solvents, and standards were purchased from Merck Company (Darmstadt, Germany).

### 2.2 Methods

#### 2.2.1 Isolation of *Lactobacilli*

Three yogurt samples were diluted using sterile distilled water to prepare dilutions ranging from 10^−1^ to 10^−5^. After dilution, the samples were cultured on MRS medium, which is specific for the growth and isolation of *Lactobacillus*, and kept for 48 h at 37°C in anaerobic jars under microaerophilic conditions (Kumar and Kumar 2014).

#### 2.2.2 Morphological and Biochemical Characteristics of Isolates

Initially, the colonies present in the culture medium were examined for color, shape, and size. The Gram staining test was then performed according to the method provided by Mannan et al. (2017). To check for the presence of the catalase enzyme, a drop of 3% hydrogen peroxide was added to a fresh culture on a sterilized glass slide and thoroughly mixed. The production of bubbles or foaming indicated a positive catalase result, while the absence of bubbles or foaming indicated a negative catalase result (Mannan et al. 2017). For the detection of the oxidase enzyme, the method of Dharmappa et al. (2022) was utilized (Dharmappa et al. 2022). Sugar fermentation tests were performed using 1% (w/v) sugar in MRS culture medium. Glucose and lactose were used as sugars and phenol red solution was used as an indicator. 10 mL of the medium was distributed, and a Durham tube was inverted in each test tube. Fresh cultures were inoculated and incubated at 37°C for 24 h. The culture medium was used as a negative control (Mannan et al. 2017).

#### 2.2.3 Screening of Biosurfactant‐Producing Bacteria

In order to isolate *Lactobacillus* bacteria according to the method of Patel et al. (2021), MRS liquid culture medium was used at 37°C (Patel et al. 2021).

##### 2.2.3.1 Hemolysis Test

To conduct the hemolysis test, the MRS broth culture of *Lactobacillus* strains was cultured on blood agar and incubated at 37°C for 72 h. Areas of clear or greenish hemolysis were considered as β and α hemolysis, respectively; the absence of any clear zones around the colonies was classified as γ hemolysis (Halder et al. 2017).

##### 2.2.3.2 Drop Collapse Test

To determine the drop collapse, the method described by Youssef et al. (2004) was employed with slight modifications. Initially, 2 μL of oil was added to each well of a 96‐well microtiter plate. Subsequently, 5 μL of the culture was added to the oil surface. The shape of the drop on the oil surface was evaluated after 1 min. In the cultures producing biosurfactants, where the droplets appeared flat, they were scored as “positive.” Conversely, in cultures where the droplets were spherical, they were scored as “negative,” indicating no biosurfactant production. A scoring system was used, where “+” indicated drop spread radius smaller than 1 cm, and “++” indicated larger than 1 cm (Youssef et al. 2004).

##### 2.2.3.3 Oil Dispersion Test

For oil dispersion determination, the method by Cornea et al. (2016) was used. Initially, 50 mL of distilled water was placed in a Petri dish, and 20 μL of vegetable oil (olive oil) was added. Then, 10 μL of crude biosurfactant dissolved in PBS was added to the surface of the oil, and the diameter of the clear zones was measured (Azarashkan et al. 2022; Cornea et al. 2016).

##### 2.2.3.4 Emulsification Index

The emulsification index (EI) was determined according to the method by Al‐Seraih et al. (2022) with slight modifications. Briefly, a mixture of 1 mL of crude biosurfactant (1 mg/mL), 4 mL of distilled water, and 6 mL of oil was vortexed vigorously (LS‐100, Labtron, Italy) for 2 min and then left at 30°C for 24 h. Tween 80 (1%) was used as a positive control. The E24% was calculated using the following equation (Al‐Seraih et al. 2022).

$$\text{Emulsification index}\;\left(\%\right)=\frac{\text{Height of formed emulsion}}{\text{Total height of the solution}}\times 100$$

#### 2.2.4 Identification of Biosurfactant‐Producing Bacterial Isolates

For the identification of strains, the method by Dubernet et al. (2002) was utilized. Initially, DNA was extracted using the phenol‐chloroform method, and to amplify the 16S rRNA gene, specific primers for *Lactobacillus* and universal primers U8F and U1390R were employed. Finally, the 16S rRNA gene sequence of the target bacteria was compared with the 16S rRNA sequences of similar bacteria in the NCBI database (Dubernet et al. 2002).

#### 2.2.5 Biosurfactant Production and Extraction

The production, extraction, and relative purification of biosurfactant were carried out over several days. Initially, bacteria were cultured in a 200 mL Erlenmeyer flask containing MRS broth and incubated for three nights at 37°C on a shaker at 150 rpm. Subsequently, the contents of the flask were centrifuged at 4°C at 14,000 rpm for 20 min to completely precipitate the bacterial cells. The resulting supernatant was then adjusted to a pH of 2 using 1 N hydrochloric acid and stored overnight at 4°C to allow the biosurfactant to precipitate. The brown precipitate containing the biosurfactant was isolated through centrifugation at 4°C at 12,000 rpm for 20 min. For the relative purification of the biosurfactant, 10 mL of a chloroform/methanol mixture in a 2:1 v/v ratio was added to the obtained precipitate and shaken at 150 rpm for 15 min at 30°C. This mixture was subsequently centrifuged again at 12,000 rpm for 20 min at 4°C. The supernatant was then placed in an oven at 40°C until completely dried. Ultimately, the biosurfactant was obtained as a white precipitate from the supernatant (Chander et al. 2012).

#### 2.2.6 Emulsification Properties of the Produced Biosurfactant in Food Models

To determine the EI of biosurfactants in the food model, equal volumes of oil (sunflower, canola, olive, and rice bran) and biosurfactants or Tween 80 were mixed for 2 min using a vortex (Kavitake et al. 2020). The emulsification activity was then measured after 24, 48, and 72 h (EI24, EI48, and EI72).

#### 2.2.7 Emulsion Stability

The stability index of the emulsions was measured according to the method of Sciarini et al. (2009) with slight modifications. The prepared emulsions were transferred to sterile graduated containers and centrifuged at 2500 rpm for 5 min at 25°C (Barzegar et al. 2023; Sciarini et al. 2009). The stability index was measured after 24, 48, and 72 h (ES24, ES48 and ES72).

#### 2.2.8 Creaming Index

To determine the creaming index (CI), each emulsion was placed in a test tube, capped to prevent evaporation, and stored in a fixed location at 25°C for 72 h (Wang et al. 2010). The CI was measured after 24, 48, and 72 h (CI24, CI48, and CI72) according to the following equation.

$$\text{Creaming index}\;\left(\%\right)=\frac{\text{Height of the serum layer}}{\text{total height of emulsion}}\times 100$$

#### 2.2.9 Oxidation of Emulsions

To determine the peroxide value (PV), the AOCS method (Cd 8–53) was employed (AOCS 2009). The level of secondary oxidation in the emulsions was also determined using the thiobarbituric acid (TBARS) test according to the methods by Ye et al. (2015).

### 2.3 Statistical Analysis

The data obtained from the experiments conducted in this study were analyzed using SPSS Statistics 27.0.1 IF026 with ANOVA in a completely randomized design. Mean comparisons were performed using Duncan's test at a confidence level of 95% (*p* < 0.05). All tests were conducted in three replicates (Vatandost et al. 2021).

## 3 Results and Discussion

### 3.1 Morphological and Biochemical Characteristics of Isolates

To initially isolate the *Lactobacillus* bacteria present in yogurt, a specific *Lactobacillus* culture medium (MRS) was utilized. A total of 19 bacterial isolates were obtained from 3 yogurt samples. The morphological and biochemical characteristics of the formed colonies are presented in Table 1. The color of these colonies ranged from white to cream. The colonies were circular and appeared in both small and large sizes. Gram staining of the bacterial isolates was performed to differentiate between Gram‐positive and Gram‐negative bacteria. Of the 19 isolates, 11 were found to be Gram‐positive. The results of the catalase test indicated that the presence of the catalase enzyme was not confirmed in 7 of these 11 isolates. Additionally, 9 out of the 19 isolated strains were oxidase‐negative. Among these 9 isolates, 7 were Gram‐positive and catalase‐negative. All isolates were able to ferment glucose, but only 7 isolates were able to ferment lactose. The fermentation seen in all isolates, except for isolate N4, was homofermentative, while isolate N4 also produced some gas along with acid. Since *Lactobacillus* are Gram‐positive, catalase‐ and oxidase‐negative bacteria that ferment glucose and lactose and form circular colonies that are white, milky to creamy in color, 7 isolates N1, N4, N7, N8, N13, and N18, exhibiting these characteristics, were considered as *Lactobacillus*.

**TABLE 1:** Morphological and biochemical characteristics of isolates isolated from yogurt.

Isolate no.	Colony color	Type of colony	Gram reaction	Catalase test	Oxidase test	Glucose fermentation	Lactose fermentation
N^1^1	White	Circular, small	+	−	−	+	+
N2	Creamy white	Circular, small	+	+	−	+	−
N3	Creamy	Circular, large	−	+	+	+	−
N4	Creamy	Circular, small	+	−	−	+	+
N5	Creamy	Circular, small	−	+	+	+	−
N6	White	Circular, large	+	+	+	+	−
N7	White	Circular, small	+	−	−	+	+
N8	White	Circular, small	+	−	−	+	+
N9	Creamy white	Circular, small	−	+	+	+	−
N10	Creamy	Circular, small	+	−	−	+	+
N11	White	Circular, large	+	+	+	+	−
N12	Creamy	Circular, large	−	+	+	+	−
N13	White	Circular, small	+	−	−	+	+
N14	Creamy White	Circular, small	−	+	+	+	−
N15	White	Circular, small	−	+	+	+	−
N16	Creamy	Circular, small	−	+	+	+	−
N17	White	Circular, large	+	+	+	+	−
N18	Creamy white	Circular, small	+	−	−	+	+
N19	Creamy	Circular, large	−	+	−	+	−

^1^ Number.

In line with the results of the current research, Kumar and Kumar (2014) collected 30 samples of milk and curd from local areas in the Solan district of Himachal Pradesh, India. The color of the colonies formed on MRS medium in their study ranged from off white, shiny white to white cream, and the isolated colonies varied in size from small to large (Kumar and Kumar 2014). Mannan et al. (2017) also isolated 25 *Lactobacillus* strains from yogurt and cheese samples and confirmed the Gram‐positive nature of these isolates, which is consistent with our research findings (Mannan et al. 2017). In another study, El‐Hosseny et al. (2025) collected twenty‐two samples of natural and locally commercial dairy products (cottage‐cheese, yogurt, and sourmilk) from markets in Egypt. In this study, out of the twenty‐two samples, 14 samples were positively identified as *Lactobacillus* species in the selective MRS environment. They examined the white, smooth, and mucoid colonies microscopically and described them as rod‐shaped. Subsequently, using the VITEK 2 C system, five isolates were identified as *Lactobacillus* species (El‐Hosseny et al. 2025).

Research has shown that lactobacilli are unable to synthesize porphyrin. This inability to produce porphyrins (e.g., heme) leads to the absence of catalase and cytochrome (without heme supplements in the growth medium) in these bacteria. Consequently, they lack an electron transport chain and rely on fermentation for energy production, a finding corroborated by Goyal et al. (2012) in their study (Goyal et al. 2012; Reddy et al. 2008). Maikhan and Mohammad‐Amin (2017) also utilized the oxidase test for the identification of *Lactobacillus* strains and isolated two strains from 28 samples of milk and yogurt. Those isolates were microaerophilic and showed negative activity in the oxidase test when inoculated with tetramethyl‐p‐phenylenediamine (Maikhan and Mohammad‐Amin 2017). In fact, the enzyme cytochrome oxidase catalyzes the transfer of electrons between electron donors in bacteria and a redox dye—tetramethyl‐p‐phenylenediamine. Ultimately, this reaction results in a color change to dark purple (Dharmappa et al. 2022). Consistent with our findings, Hedberg et al. (2008) reported that the bacterium *L. plantarum* 299v was able to ferment glucose and lactose under both anaerobic and atmospheric conditions (with 5% carbon dioxide) in their study (Hedberg et al. 2008). Forouhandeh et al. (2010) isolated LAB from various traditional and local cheeses and yogurts, and the biochemical characteristics of all isolates were tested using carbon sources. During glucose fermentation, acid production was reported in the Durham tube (Forouhandeh et al. 2010).

### 3.2 Screening of Biosurfactant‐Producing Bacteria

#### 3.2.1 Hemolysis Test

To evaluate hemolytic activity, 7 *Lactobacillus* strains were cultured on blood agar medium. None of these 7 isolates exhibited the ability to cause hemolysis in this medium and were unable to break down red blood cells or clear the agar. In fact, biosurfactants can cause the lysis of red blood cells, resulting in the formation of a clear and colorless zone around the colonies. Given that no clear zone was observed around the colonies, γ‐hemolysis was considered. Numerous studies have shown that biosurfactants from *Lactobacillus* species do not possess the ability to induce hemolysis in blood agar. In accordance with the present study, Talib et al. (2019) investigated the hemolytic activity of 10 *Lactobacillus* isolates obtained from Malaysian kefir grains. They reported that none of the isolates showed hemolysis on blood agar, and all isolates were γ‐hemolytic (Talib et al. 2019). Halder et al. (2017) isolated *Lactobacillus* strains *L. animalis* LMEM6, *L. plantarum* LMEM7, *L. acidophilus* LMEM8, and *L. rhamnosus* LMEM9 from four samples of curd. They observed no clear or green zones around their colonies on blood agar (Halder et al. 2017). Meng et al. (2021) also reported that the strains *L. plantarum* AHQ‐14 and *L. bulgaricus* BD0390 in their study did not produce clear zones on blood agar and were identified as γ‐hemolytic bacteria. Many researchers (Jin et al. 2019; Kim et al. 2018; Yasmin et al. 2020) have reported that probiotics do not exhibit hemolytic activity (Meng et al. 2021). Suwanwong (2004) also believed that extracellular enzymes and biosurfactants are effective in the lysis of blood cells. However, blood agar is a complex medium and can sometimes complicate the detection of surfactants, particularly when microorganisms are examined directly on the agar (Suwanwong 2004). The lack of hemolysis of red blood cells may also result from limited diffusion of surfactants in the agar‐containing medium. Therefore, in the subsequent part of this study, the ability to produce biosurfactants was investigated using a more precise method.

#### 3.2.2 Droplet Collapse Test

The droplet collapse test is one of the methods for detecting biosurfactant production, developed by Jain et al. (1991). The results of the droplet collapse test are presented in Table 2. According to the results, the control sample (distilled water) did not collapse in the paraffin layer of the well and remained on the surface like a bead. In contrast, Tween 80 collapsed within about 1 min and moved to the bottom of the well. A similar observation was made for the bacterial suspension, indicating biosurfactant production by the bacteria. The highest droplet collapse was recorded in isolates N4, N10, and N18. Tween 80 (a chemical surfactant) also demonstrated the highest droplet collapse activity, similar to the mentioned isolates. Generally, when a liquid contains a surfactant, droplets spread or collapse due to the reduced interfacial tension between the liquid droplet and the hydrophobic surface. In a study conducted by Patel et al. (2021), the capability of biosurfactant production by *L. rhamnosus* was evaluated using the drop collapse test. They reported that this bacterium had performed positively in that assay and had been capable of producing biosurfactants (Patel et al. 2021). Kaur et al. (2015) also utilized the droplet collapse method for screening biosurfactant‐producing bacteria. They isolated 19 LAB strains and observed no activity for distilled water. However, the supernatant droplets from 14 isolates (73%) resulted in drop collapse, indicating a reduction in surface activity and biosurfactant production. Consequently, the selected 14 isolates underwent further investigation using the oil spreading test (Kaur et al. 2015).

**TABLE 2:** The results of drop collapse test.

Sample	Drop collapse
N^1^1	+
N4	++
N7	+
N8	+
N10	++
N13	+
N18	++
Tween 80	++
Control	−

*Note:* + to show drops with a diameter smaller than 1 cm; ++ to show drops with a diameter larger than 1 cm.

^1^ Isolate number.

#### 3.2.3 Oil Spreading Test

The oil spreading test is a simple and rapid method used to detect biosurfactant production. This method is based on the reduction of interfacial tension between water and oil, resulting in the formation of a clear zone due to effective biosurfactants (Essghaier et al. 2023). The results obtained from the oil spreading test are presented in Table 3. In this study, Tween 80 and distilled water exhibited the highest and lowest surface activity, respectively. All isolates demonstrated varying surface activities, spreading oil from 2.9 ± 0.08 to 8.1 ± 0.16 cm (*p* < 0.05). This result indicates the presence of a biosurfactant in the culture medium of *Lactobacillus* bacteria. The lowest surface activity observed among the isolates was associated with the biosurfactant derived from isolate N10. Although the mechanism of oil spreading by biosurfactants is not fully understood at the molecular level, it is considered a sensitive and simple method for measuring the surface‐active nature of biosurfactants (Kaur et al. 2015). The presence of biosurfactants in the supernatant liquid causes oil to be displaced on the water surface, which occurs due to the amphiphilic properties of the biosurfactants (Madhu and Prapulla 2014). In agreement with the present study, Chigede et al. (2024) employed the oil‐spreading technique to identify biosurfactant‐producing bacteria. They reported that, unlike the bacterial culture supernatant, distilled water did not exhibit any cleaning activity (Chigede et al. 2024). Cornea et al. (2016) used the oil spreading test to screen for biosurfactant‐producing strains among 10 *Lactobacillus* strains. Their research results indicated that at least three *Lactobacillus* strains (L26, L35, and L61) were capable of biosurfactant production. These three *Lactobacillus* strains (L26, L35, and L61) were selected for further analysis. They reported that strains L26 and L35 belonged to *L. plantarum* , while strain L61 was identified as *L. brevis* (Cornea et al. 2016). Lara et al. (2022) reported isolating 9 *Lactobacillus* strains from sheep cheese and fish ( *Odontesthes bonariensis* ) and stated that all of them produced cell‐associated biosurfactants. The diameter of the clear zone created by the biosurfactant produced from the strains *Lactobacillus plantarum* (LbTw3), *L. plantarum* (LbTw5), and *Lactobacillus paraplantarum* (LbTw7) in sheep cheese, as well as from the strains *Lactococcus lactis ssp. lactis* (Tw12), *L. lactis ssp. lactis* (Tw34), *L. lactis ssp. lactis* (Tw35), *Lactobacillus pentosus* (Tw226), and *Leuconostoc mesenteroides ssp. jonggajibkimchii* (Tw234) in fish were reported to be 1, 1, 1.8, 1.2, 1.8, 1, 1.8, 1.8, and 1.6 cm, respectively (Lara et al. 2022).

**TABLE 3:** The results of oil spreading test.

Sample	Clear zone diameter (cm)
N^1^4	8.1 ± 0.16^b^
N10	2.9 ± 0.08^d^
N18	7.4 ± 0.04^c^
Tween 80	9.3 ± 0.07^a^
Control	0

*Note:* Mean values within the same column not sharing a common superscript letters differ significantly (*p* < 0.05).

^1^ Isolate number.

#### 3.2.4 Emulsification Index

The EI is a common method based on the emulsification capacity of biosurfactants. This index is calculated based on the ratio of the height of the emulsified layer to the total height of the mixture (Walter et al. 2010). The results obtained from the measurement of the EI are summarized in Table 4. In this study, the EI of the chemical surfactant Tween 80 (positive control) was determined to be 64.36%. Isolates N4 and N18 also exhibited high EI, making them suitable candidates for biosurfactant production. Consequently, these two isolates were selected for bacterial identification and biosurfactant extraction. According to the present study, researchers such as Madhu and Prapulla (2014), Al‐Seraih et al. (2022), Essghaier et al. (2023), and Chigede et al. (2024) have investigated the EI of microbial biosurfactants (Al‐Seraih et al. 2022; Chigede et al. 2024; Essghaier et al. 2023; Madhu and Prapulla 2014). Furthermore, Patel et al. (2021) reported that the emulsification activity of the biosurfactant produced from *Lactobacillus rhamnosus* ‐MBP002 in their research was 32.37% (Patel et al. 2021).

**TABLE 4:** The results of emulsification index (%).

Sample	EI (%)
N^1^4	42.49 ± 0.81^b^
N10	20.87 ± 1.62^d^
N18	39.05 ± 0.32^c^
Tween 80	64.36 ± 0.45^a^
Control	0

*Note:* Mean values within the same column not sharing a common superscript letters differ significantly (*p* < 0.05).

^1^ Isolate number.

### 3.3 Identification of Biosurfactant‐Producing Bacterial Isolates

Isolates N4 and N18, based on the results presented in Tables 2, 3, 4 regarding the oil spreading, drop collapse, and EI tests, demonstrated excellent performance. Therefore, the 16S rDNA gene sequence of these bacteria was determined. The results obtained from the PCR of the 16S rDNA gene using *Lactobacillus*‐specific primers, including LBLMA1 and R16‐1, confirmed the presence of a band with a size of 220 base pairs on a 1% agarose gel, indicating that the bacteria isolated from local dairy products belonged to the genus *Lactobacillus*. After amplifying the 16S rDNA gene with universal primers U8F and U1390R, the PCR product measuring 1400 base pairs was sequenced. The alignment of the 16S rDNA gene sequence showed that isolate N4 was closely related to *L. plantarum* LBp_WAM with 99% similarity, while isolate N18 was closely aligned with *L. rhamnosus* DSA LR1 with 98% similarity.

### 3.4 Emulsification Properties of the Produced Biosurfactant in Food Models

Emulsions are complex two‐phase systems formed by droplets dispersed in a continuous phase. Surfactants are essential for facilitating the formation of an emulsion by positioning two immiscible substances together. Generally, a good biosurfactant creates a relatively high EI (Ashaolu and Zhao 2020). According to Table 5, the EI increased after the extraction of biosurfactants from isolates N4 and N18. Additionally, both the biosurfactants and Tween 80 effectively stabilized emulsions formed from olive and canola oils. The EI of each of these surfactants in creating emulsions with sunflower oil was also high; however, the lowest EI of the biosurfactants was observed in rice bran oil. Furthermore, the EI for each emulsifier decreased over time in various oils. The type of surfactant significantly influenced the EI. For instance, the highest EI was initially observed in samples where Tween 80 was used as the surfactant. Additionally, the biosurfactant derived from the *L. plantarum* strain exhibited a higher EI than the biosurfactant obtained from the *L. rhamnosus* strain across all samples. A higher EI indicates a greater efficacy of this surfactant in reducing the interfacial tension between two immiscible liquids and in creating a stable emulsion. The EI of microbial biosurfactants in oily environments has been investigated in various studies (Ashaolu and Zhao 2020; Madhu and Prapulla 2014; Ribeiro et al. 2020). In line with the present study, Cornea et al. (2016) measured the EI of biosurfactants produced by *L. brevis* and *L. plantarum* in sunflower and olive oils. They reported that the EI in olive oil was higher than in sunflower oil for all strains (Cornea et al. 2016).

**TABLE 5:** The results of examining the emulsification index (%) of emulsions prepared with biosurfactants obtained from *L. plantarum* and *L. rhamnosus* and Tween 80 chemical surfactant.

Oil type	Surfactant type								
	BS^1^ of *L. plantarum*			BS of *L. rhamnosus*			Tween 80		
	E_24_	E_48_	E_72_	E_24_	E_48_	E_72_	E_24_	E_48_	E_72_
Olive oil	52.83 ± 1.77^a^	52.79 ± 3.18^a^	46.25 ± 0.86^a^	54.07 ± 0.81^a^	51.82 ± 0.41^a^	49.57 ± 1.46^a^	59.05 ± 1.14^a^	58.79 ± 0.18^a^	56.38 ± 0.94^a^
Rice bran oil	39.51 ± 2.44^b^	33.12 ± 3.34^c^	30.34 ± 2.53^c^	39.78 ± 1.45^b^	33.64 ± 1.63^b^	29.02 ± 0.89^d^	51.27 ± 1.63^b^	49.38 ± 0.99^c^	44.42 ± 0.5^b^
Canola oil	50.75 ± 0.61^a^	46.89 ± 3.15^ab^	43.72 ± 2.28^ab^	50.52 ± 1.17^a^	49.98 ± 0.81^a^	45.63 ± 1.63^b^	58.32 ± 0.65^a^	55.46 ± 0.38^b^	55.17 ± 0.11^a^
Sunflower oil	43.11 ± 1.54^b^	39.76 ± 3.23^bc^	39.02 ± 2.44^b^	40.07 ± 2.41^b^	39.67 ± 0.62^c^	37.33 ± 0.97^c^	58.51 ± 0.42^a^	56.43 ± 1.06^b^	55.29 ± 0.73^a^

*Note:* Mean values within the same column not sharing a common superscript letters different significantly (*p* < 0.05).

Abbreviations: E24, emulsification index after 24 h; E48, emulsification index after 48 h; E72, emulsification index after 72 h.

^1^ Biosurfactant.

### 3.5 Emulsion Stability

**FIGURE 1:**
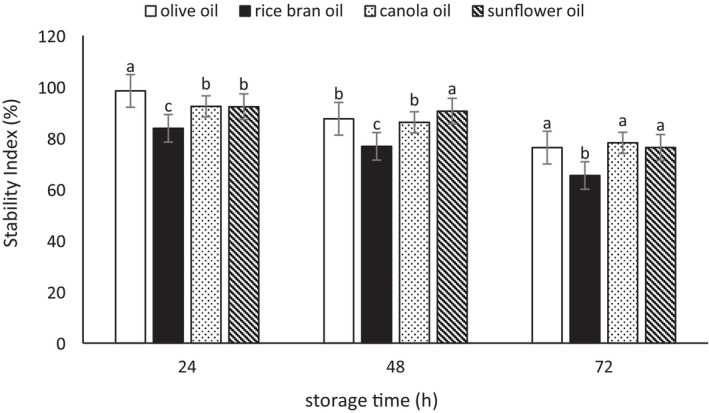
The stability index (%) of the emulsions prepared with the biosurfactant from *L. plantarum* . Mean values at a constant time that do not share a common superscript are significantly different (*p* < 0.05).

**FIGURE 2:**
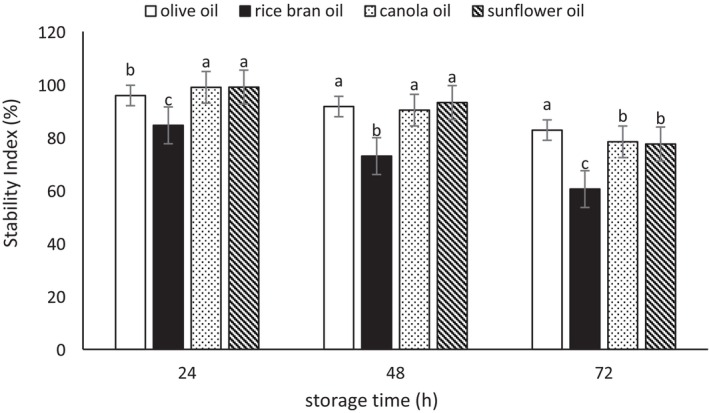
The stability index (%) of the emulsions prepared with the biosurfactant from *L. rhamnosus* . Mean values at a constant time that do not share a common superscript are significantly different (*p* < 0.05).

**FIGURE 3:**
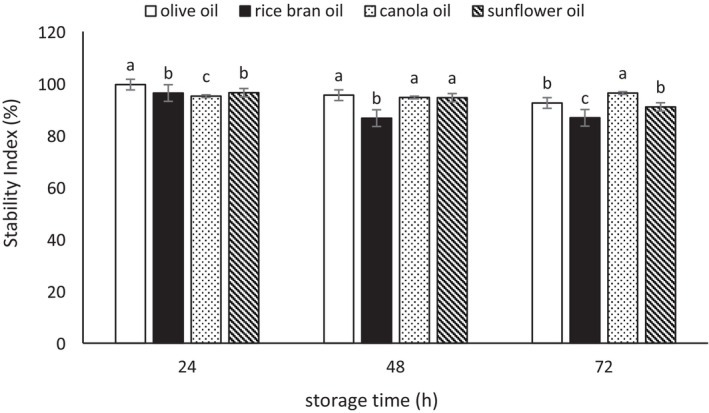
The stability index (%) of the emulsion prepared with Tween 80. Mean values at a constant time that do not share a common superscript are significantly different (*p* < 0.05).

The results of the evaluation of emulsion stability (ES) index are presented in Figures 1, 2, 3. According to the three mentioned figures, the emulsions showed considerable stability after 3 days. During the 24 h, the stability level of the emulsions was very high; however, over time, the stability of the emulsions decreased. The highest stability was observed in the emulsions prepared with Tween 80 (86.76%–96.31% at 72 h of storage), followed by the biosurfactant derived from *L. plantarum* (65.4%–78.16% at 72 h of storage). Furthermore, the highest ES was recorded in the emulsions formed with olive oil, while the least stability pertained to the emulsion prepared with rice bran oil. In general, the reduction in the EI over time leads to a decrease in ES (Zhang et al. 2022). The stability of the emulsion is determined by dividing the final height of the emulsion by the initial height of the emulsion. This means that the lower the final height of the emulsion or the greater the initial height compared to the final height, the lower the stability of the emulsion. Additionally, several factors such as storage conditions, type of oil, characteristics of the emulsifier, and chemical factors (such as oxidation) can significantly influence EI and ES (Ribeiro et al. 2020). In a study, the creation of stable systems was linked to the ability of surfactants to delay the increase in droplet size by aggregation (Grit and Crommelin 1993). Prasanna et al. (2012) also provided similar reports confirming the reduction of ES over time (Prasanna et al. 2012; Ghorbani‐HasanSaraei et al. 2019).

### 3.6 Creaming Index

**FIGURE 4:**
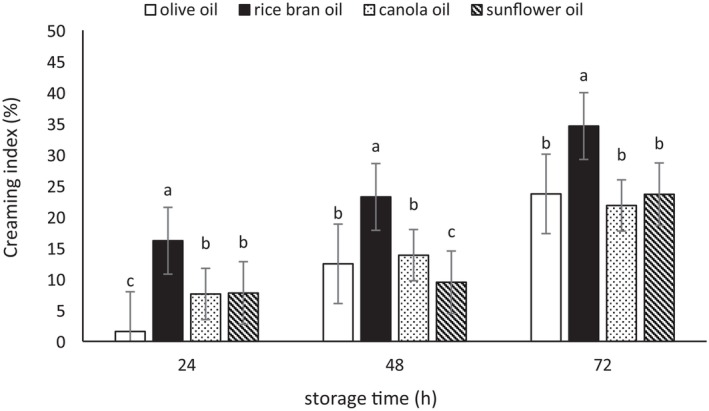
The creaming index (%) of the emulsions prepared with the biosurfactant from *L. plantarum* . Mean values at a constant time that do not share a common superscript are significantly different (*p* < 0.05).

**FIGURE 5:**
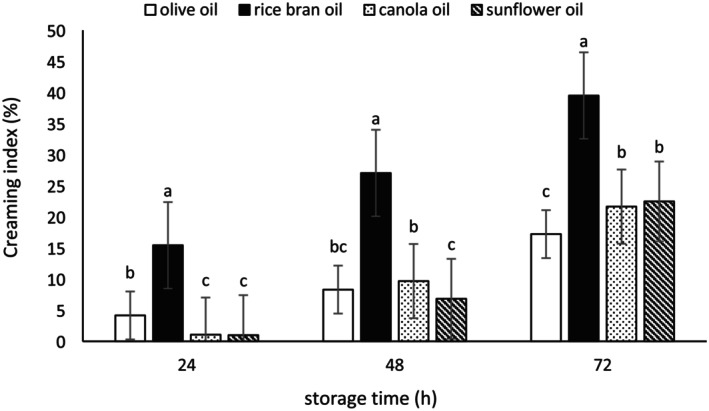
The creaming index (%) of the emulsions prepared with the biosurfactant from *L. rhamnosus* . Mean values at a constant time that do not share a common superscript are significantly different (*p* < 0.05).

**FIGURE 6:**
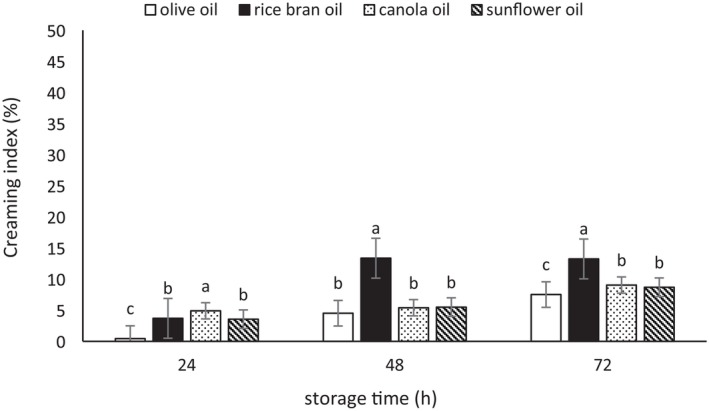
The creaming index (%) of the emulsion prepared with Tween 80. Mean values at a constant time that do not share a common superscript are significantly different (*p* < 0.05).

Emulsions may become unstable over time due to various physicochemical processes, such as gravitational separation, coalescence, and aggregation. One important test for determining the stability of emulsions is the measurement of the CI (Gardouh et al. 2012; Wangpradit et al. 2022). The results of the CI measurements for the emulsions are presented in Figures 4, 5, 6. These three figures showed an increasing trend in CI as storage time progressed. The CI of the emulsions prepared with Tween 80 was lower compared to those produced with microbial biosurfactants derived from *L. plantarum* and *L. rhamnosus* . The highest and lowest CI values were observed for emulsions prepared with rice bran oil and olive oil, respectively (*p* < 0.05). Furthermore, no significant difference in the CI was observed between canola oil and sunflower oil. In general, the greater the stability of an emulsion, the lower the phase separation and CI. Various factors, such as the type of oil, storage conditions, the type and chemical characteristics of the emulsifier, oxidation, and changes in the physical properties of the emulsifier, can significantly influence EI and ES, and thereby affect the CI as well (Ribeiro et al. 2020). Kampa et al. (2022) concluded that the type of oil has a significant impact on the CI values. Contrary to the findings of this study, the CI in the emulsion prepared with olive oil was slightly higher than that of the sunflower oil. They explained that this difference could be attributed to factors such as droplet size and the density of the dispersed phase. Furthermore, they stated that Stokes' law could confirm that the rate of gravitational separation can be reduced by decreasing droplet size, lowering the density difference between the dispersed and continuous phases, and increasing the viscosity of the continuous phase (Kampa et al. 2022).

### 3.7 Oxidation of Emulsions

The potential antioxidant effect of biosurfactants produced from two *Lactobacillus* strains and Tween 80 in canola and olive oil was investigated due to the high EI of these surfactants in these oils, their high stability, and lower creaming compared to the other two oils, using PV and thiobarbituric acid (TBARS) tests. The determination of PV is one of the most common chemical tests indicating the concentration of hydroperoxides produced during the early stages of oxidation (Zhang et al. 2021). Results regarding the initial oxidation of emulsions prepared with olive and canola oil over the storage period are presented in Tables 6 and 7, respectively. As seen, the PV levels formed in all samples have shown an increasing trend during the storage period, with this increase being higher in the control sample than in the others. The lowest PV level at 72 h of storage was also observed in the emulsions prepared with Tween 80 (2.29 ± 0.28 and 2.31 ± 0.11 meq O_2_/kg for the emulsions made with olive oil and canola oil, respectively).

**TABLE 6:** The results of measuring the peroxide value (meq O_2_/kg) of emulsions prepared with olive oil.

Surfactants	Storage time (h)			
	0	24	48	72
BS^1^ *L. plantarum*	0.34 ± 0.008^a^	1.02 ± 0.15^b^	2.25 ± 0.14^b^	3.11 ± 0.18^b^
BS *L. rhamnosus*	0.36 ± 0.011^a^	1.17 ± 0.14^ab^	2.43 ± 0.16^b^	3.08 ± 0.16^b^
Tween 80	0.38 ± 0.007^a^	0.85 ± 0.12^b^	1.42 ± 0.08^c^	2.29 ± 0.28^c^
Control	0.33 ± 0.020^a^	1.44 ± 0.13^a^	2.96 ± 0.04^a^	4.23 ± 0.20^a^

*Note:* Mean values within the same column not sharing a common superscript letters differ significantly (*p* < 0.05).

^1^ Biosurfactant.

**TABLE 7:** The results of measuring the peroxide value (meq O_2_/kg) of emulsions prepared with canola oil.

Surfactants	Storage time (h)			
	0	24	48	72
BS^1^ *L. plantarum*	0.39 ± 0.04^a^	1.15 ± 0.03^b^	2.57 ± 0.37^b^	3.38 ± 0.04^b^
BS *L. rhamnosus*	0.44 ± 0.03^a^	1.24 ± 0.05^b^	2.49 ± 0.16^b^	3.40 ± 0.08^b^
Tween 80	0.42 ± 0.07^a^	1.01 ± 0.03^c^	1.63 ± 0.10^c^	2.31 ± 0.11^c^
Control	0.41 ± 0.09^a^	1.76 ± 0.06^a^	3.26 ± 0.33^a^	4.79 ± 0.06^a^

*Note:* Mean values within the same column not sharing a common superscript letters differ significantly (*p* < 0.05).

^1^ Biosurfactant.

The higher control of oxidation in emulsions prepared with Tween 80 compared to biosurfactants may be attributed to the superior emulsifying capacity of Tween 80, leading to better formation and stability of the emulsions. In fact, a high emulsifying capacity indicates increased stability of the system and prevents phase separation, which can contribute to a reduced rate of oil oxidation (McClements et al. 2017). Therefore, in this study, both Tween 80 and subsequently the biosurfactants positively influenced the prevention of oil oxidation.

**FIGURE 7:**
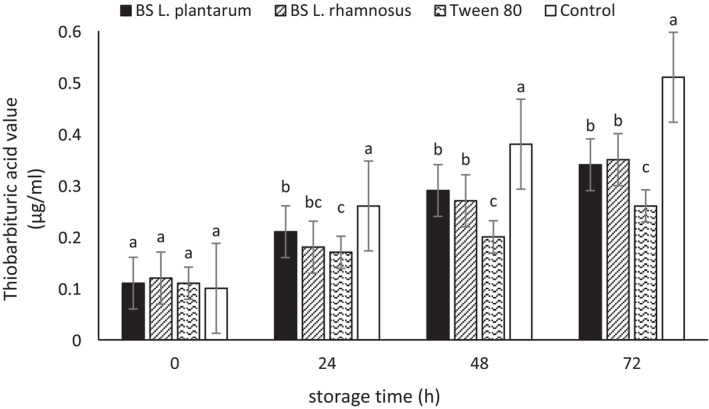
TBARS value emulsions prepared with olive oil. BS *L. plantarum* , biosurfactant produced from *L. plantarum* ; BS *L. rhamnosus* , biosurfactant produced from *L. rhamnosus* . Mean values at a constant time that do not share a common superscript are significantly different (*p* < 0.05).

**FIGURE 8:**
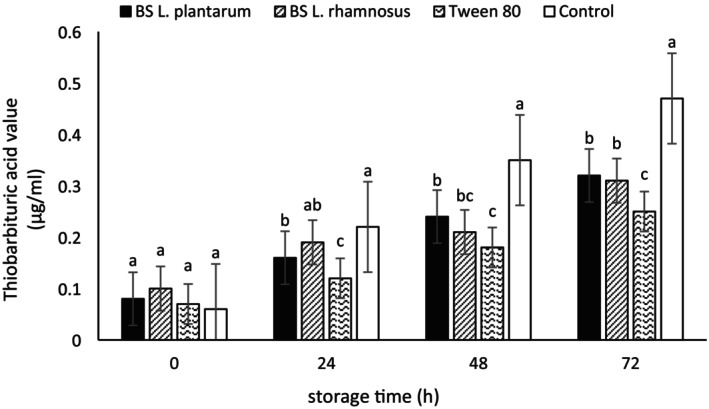
TBARS value of emulsions prepared with canola oil. BS *L. plantarum* , biosurfactant produced from *L. plantarum* ; BS *L. rhamnosus* , biosurfactant produced from *L. rhamnosus* . Mean values at a constant time that do not share a common superscript are significantly different (*p* < 0.05).

The TBARS test is one of the common methods for determining secondary oxidation products (Ghelichi et al. 2023). As shown in Figures 7 and 8, the TBARS levels were low in the initial hours across all samples, with no significant differences among samples. Over time, the primary oxidation products were degraded, leading to an increase in this index (Kong and Singh 2016). In the current study, the highest TBARS levels were observed in the control sample. Emulsions prepared with microbial biosurfactants exhibited lower TBARS levels compared to the control sample. Furthermore, the type of biosurfactant had no significant effect on the TBARS levels of each of the prepared emulsions. However, the lowest TBARS was recorded in the sample containing Tween 80. In line with the present study, Di Mattia et al. (2014) reported that the control sample in their research exhibited a greater increase in PV over time compared to other samples that utilized surfactants (Di Mattia et al. 2014). Additionally, Nollet and Toldrá (2011) indicated that an acceptable range for TBARS in fat products is approximately 1 mg MDA/kg (Nollet and Toldrá 2011). He et al. (2017) highlighted the positive effect of using biosurfactants in enhancing the oxidative stability of microemulsions (He et al. 2017).

Research has shown that biosurfactants derived from *lactobacilli* consist of glycoproteins and possess antioxidant properties. The amino acids present in the protein portion can function as chelating agents for metal ions, unpaired electrons, and free radicals, which play a significant role in the ability of peptides to reduce free radicals (Hu et al. 2023). Therefore, the lower level of oxidation observed in the emulsions prepared with biosurfactants compared to the control sample can likely be attributed to the role of the glycolipid structure in reducing the oxidation rate. Additionally, biosurfactants, due to their high emulsifying index, were able to create stable emulsions, which consequently prevented phase separation and may lead to a reduced rate of oil oxidation.

## 4 Conclusion

The increasing awareness in society regarding the harmful effects of synthetic surfactants has heightened consumer willingness to use natural microbial surfactants. The results of this study demonstrated that the biosurfactants derived from *L. plantarum* LBp_WAM and *L. rhamnosus* DSA LR1 produced stable emulsions in olive oil, canola oil, sunflower oil, and rice bran oil. However, their effectiveness was lower than that of the synthetic surfactant Tween 80. The biosurfactants obtained from these two strains exhibited higher emulsification and stability indices in olive oil and canola oil compared to sunflower oil and rice bran oil. Furthermore, the assessment of oxidation levels revealed that these biosurfactants possessed antioxidant properties, reducing the oxidation rate relative to the control sample. The present study confirmed that the biosurfactant produced by *L. plantarum* and *L. rhamnosus* has a high capacity to form stable emulsions with olive and canola oils.

## Author Contributions

**Roghayeh Rezaeimalidareh:** formal analysis (equal), methodology (equal), software (equal), visualization (equal), writing – original draft (equal). **Mohammad Ahmadi:** investigation (equal), resources (equal), supervision (equal), writing – review and editing (equal). **Seyed‐Ahmad Shahidi:** conceptualization (equal), project administration (equal), supervision (equal), writing – review and editing (equal).

## Conflicts of Interest

The authors declare no conflicts of interest.

## Data Availability

The authors have nothing to report.
